# Enhancing surgical precision and efficiency: a study and comparison of a three-dimensional surgical video system in proliferative diabetic retinopathy surgery

**DOI:** 10.3389/fmed.2023.1246936

**Published:** 2023-10-02

**Authors:** Xiang Zhang, Danni Zhu, Wenbo Li, Hanling Hu, Zetong Nie, Haoxin Guo, Zhaoxiong Wang, Xiaorong Li, Bojie Hu

**Affiliations:** Department of Ophthalmology, Eye Institute, Tianjin Medical University Eye Hospital, Tianjin Medical University Eye Institute, Tianjin, China

**Keywords:** 3D surgical video system, vitreoretinal surgery, proliferative diabetic retinopathy (PDR), diabetics, retinopathies

## Abstract

**Purpose:**

This study aimed to investigate the safety and efficacy of three-dimensional (3D) surgical video systems for proliferative diabetic retinopathy (PDR).

**Methods:**

This retrospective clinical case study included 30 patients (30 eyes) with PDR. Patients were divided into two groups: one underwent surgery using a 3D surgical video system (14 cases, 14 eyes), while the other underwent traditional microscope surgery (16 cases, 16 eyes). Safety and efficacy were assessed through predetermined surgical parameters, including surgical duration, intraoperative membrane removal rate, and occurrences during intraoperative and postoperative phases.

**Results:**

Our study revealed noteworthy differences in various aspects between the 3D surgical video system group and the traditional microscope surgery group. Specifically, the mean surgical time was 30.25 ± 14.43 mins in the 3D surgical video system group, while it was 38.56 ± 18.71 mins in the traditional microscope surgery group (*p* = 0.051). Furthermore, the mean membrane removal time was significantly shorter in the 3D group at 2.53 ± 1.52 mins, as compared to 3.23 ± 1.76 mins in the traditional group (*p* = 0.042). Importantly, the membrane removal rate also displayed a significant difference, with the 3D group at 0.55 ± 0.07 and the traditional group at 0.41 ± 0.11 (*p* = 0.018). However, no notable differences were observed between the two groups in terms of intraoperative and postoperative incidences.

**Conclusion:**

The safety and efficacy obtained using the 3D surgical video system in PDR surgery were comparable to those obtained in traditional microscopic surgery.

## 1. Introduction

Proliferative diabetic retinopathy (PDR), a severe threat to vision, affects ~6.96% of patients with diabetes globally, making it the leading cause of blindness in the working-age population (1–3). PDR is characterized by the formation of neovascularized and proliferative membranes, which can lead to vitreous hemorrhage, tractional retinal detachment, neovascular glaucoma, and other serious consequences (4–6). Minimally invasive vitrectomy is widely employed for treating PDR, as it effectively clears vitreous hemorrhage and removes the tractional effects caused by proliferative membranes. However, during surgery, unclear visualization of the intraocular tissue layers often necessitates repetitive instrument manipulation, resulting in prolonged surgical time and increased risks of iatrogenic complications, such as retinal damage, recurrent vitreous hemorrhage, and retinal detachment. Therefore, efficient and safe completion of the surgery, along with a reduction in the complication risk, is crucial for patients with severe PDR (7–10).

In recent years, three-dimensional (3D) display technology has undergone rapid advancements and has been extensively applied in ophthalmic surgeries requiring high precision and stability. Three-dimensional (3D) surgical video systems are equipped with advanced components, such as a 3D, 4K ultra-high-definition organic light-emitting diode (OLED) 55-inch display screens and high dynamic range (HDR) cameras. These features provide ophthalmic surgeons with a high-definition stereoscopic surgical view and precise spatial orientation. Compared with traditional surgery performed under a microscope, 3D surgical video systems offer significant advantages, such as increased depth of field, higher resolution, reduced retinal phototoxicity, ergonomic design, and support for teaching and instruction. Domestic and international studies have demonstrated the widespread application of 3D surgical video systems in surgeries, such as cataract surgery, macular hole surgery, epiretinal membrane surgery, and macular pucker surgery (11–14). However, for the complex intraocular conditions present in patients with PDR, further investigation is required to evaluate the safety and effectiveness of 3D surgical video systems in their surgeries.

Based on the aforementioned background, our center conducted a retrospective study to evaluate the safety and efficacy of the 3D surgical video system in PDR surgeries, as compared to the traditional microscope method.

## 2. Methods

### 2.1. Study design

This is a retrospective, controlled study.

### 2.2. Participants

This study was approved by the Ethics Committee of Tianjin Medical University Eye Hospital (Approval No. 2022JS-01). All patients were informed of the study's purpose and provided signed consent in accordance with the principles of the Declaration of Helsinki. This single-center retrospective case study aimed to analyze the clinical data of 30 patients (30 eyes) diagnosed with PDR, who underwent minimally invasive vitrectomy at Tianjin Medical University Eye Hospital in Tianjin, China, between April and October 2022.

The inclusion criteria were patients diagnosed with PDR who required surgical treatment owing to the presence of vitreous/retinal hemorrhage and/or tractional retinal detachment confirmed by fundus imaging or ocular B-scan.

The exclusion criteria were as follows: (1) corneal opacities or corneal opacities that affected visual evaluation; (2) other vitreoretinal or ocular congenital diseases involving the iris, choroid, or optic nerve; (3) vitreous hemorrhage, retinal detachment, or other conditions unrelated to diabetic retinopathy requiring vitrectomy surgery; (4) previous vitreoretinal surgery; and (5) difficulties in follow-up or inability to complete follow-up.

Participants in this study were categorized into two distinct groups based on the observation equipment utilized during the surgical procedure. The first group, referred to as the “3D Surgical Video Group,” comprised patients who underwent surgery with the assistance of the 3D surgical video system. The second group, designated as the “Traditional Microscope Group,” consisted of patients who underwent surgery using the traditional microscope system.

Preoperative best-corrected visual acuity (BCVA), intraocular pressure (IOP), slit-lamp examination, indirect ophthalmoscopy, and ocular B-scan results were recorded using an electronic medical record system. The BCVA was measured using the international standard visual acuity chart and converted to the logarithm of the minimum angle of resolution (logMAR). The results were manually converted to 2.3 or 2.0 logMAR. Noncontact tonometry was performed to measure IOP in postoperative follow-up examinations involving several aspects, including postoperative BCVA and IOP. The examination also aimed to detect instances of IOP, vitreous hemorrhage, or retinal detachment after surgery.

### 2.3. Surgical procedure

**Figure 1:**
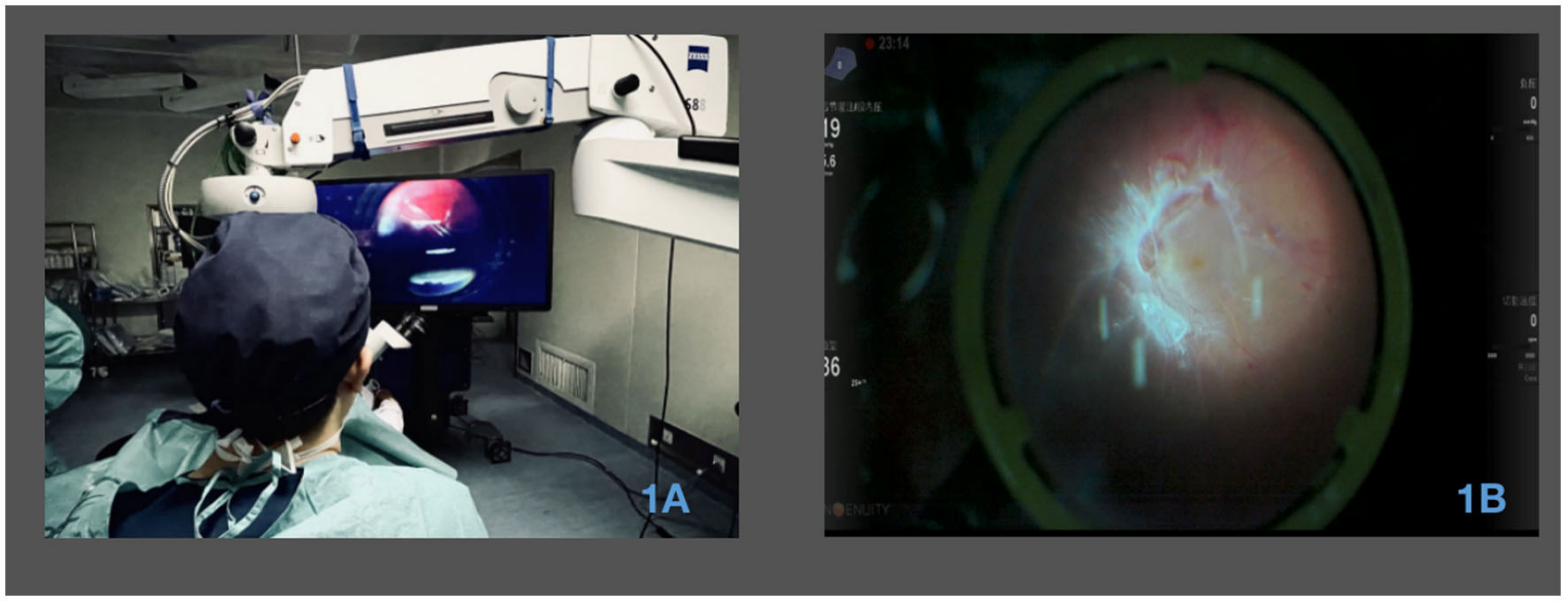
Utilization of a 3D surgical video system in PDR vitrectomy surgery. **(A)** Surgical perspective with 3D visualization during PDR vitrectomy surgery. The image showcases the view seen by the surgeon through the 3D visualization system during the surgical procedure. **(B)** Utilization of 3D visualization for membrane peeling during surgery. The image illustrates the application of the 3D visualization system for the process of membrane peeling during the surgical procedure.

All surgeries were performed by a single experienced vitreoretinal surgeon. It is noteworthy that the surgeon performing the procedures in the “3D Surgical Video Group” had extensive prior experience with 3D surgical video systems, having successfully completed hundreds of surgeries using this technology before the study's commencement. This surgeon's familiarity with the nuances of the 3D system may have impacted certain aspects of the surgeries and potentially influenced the outcomes evaluated. A traditional microscope system (OPMI Lumera T; Carl Zeiss AG, Jena, Germany) was used in the “Traditional Microscope Group,” while a 3D surgical video system was used in the 3D group (NGENUITY; Alcon Inc., Fort Worth, TX, USA). The 3D display screen was placed ~1.2–1.8 m in front of the surgeon, and the surgeon, assistant, and observers were required to wear 3D polarized glasses (Figure 1). In both groups, a Resight500 non-contact wide-angle lens (Zeiss, Oberkochen, Germany) was used to observe the fundus, and the surgery was performed using the Constellation vitrectomy system under the same magnification. Three days prior to surgery, the patients were administered moxifloxacin eye drops and levofloxacin eye ointment. Intraoperatively, 5% povidone-iodine was used for ocular disinfection, and standard three-port pars plana vitrectomy was performed under local anesthesia with an injection of 2 mL of 2% lidocaine and 2 ml of 0.1% bupivacaine posterior to the globe. The decision to remove the lens was based on the surgeon's assessment of the degree of opacification, its impact on visual function, and intraoperative manipulation. The IOP was maintained at 20–30 mmHg during surgery. The vitreous hemorrhage was removed, and the vitreous and posterior cortical vitreous were cleared by applying pressure and removing the basal vitreous. For patients with proliferative membranes and tractional retinal detachment, the main goal was to relieve traction, and methods such as cutting, layering, and peeling were employed to completely remove the fibrovascular membranes. If there was tight adhesion between the membranes and retinal vessels, a high-speed vitrectomy cutter was used to tear the membranes to avoid retinal tears or bleeding caused by improper manipulation. Hemostasis was achieved by diathermy, followed by gas-fluid exchange and panretinal laser photocoagulation. Silicone oil or C3F8 gas was added, if necessary, to reposition the retina.

### 2.4. Intraoperative quantitative measures

We collected data on several parameters during surgical interventions for PDR to assess surgical efficiency and patient outcomes. These measures included the following:

#### 2.4.1. Total surgical time

The duration of the entire surgical procedure was recorded in minutes, from the establishment of the three-port sclerotomy to the removal of the trocar cannulas and closure of the surgical incisions. This information was obtained from video recordings of the surgeries.

#### 2.4.2. Membrane removal time

The time taken to completely remove the proliferative membranes was recorded in minutes. This measurement began with the initial use of a vitreous cutter or intraocular forceps to cut, grasp, and separate the membranes and concluded when all the membranes were completely excised. These timings were derived from surgical videos.

#### 2.4.3. Membrane removal rate

**Figure 2:**
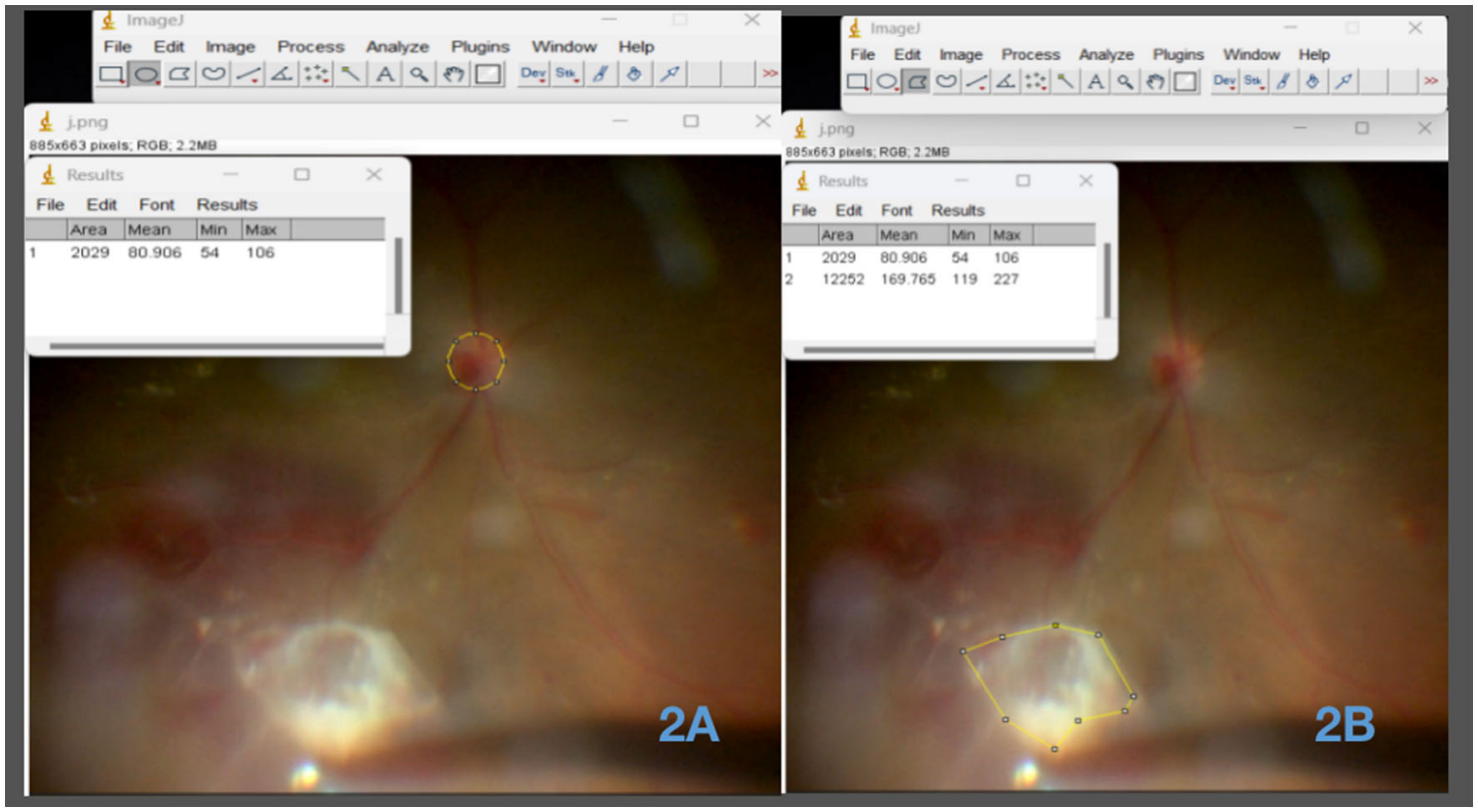
Circumferential analysis of the optic disk and neovascular membrane areas using ImageJ software. **(A)** Measurement of the optic disk area using ImageJ software. This image depicts the utilization of ImageJ software to accurately measure and outline the area of the optic disc. **(B)** Measurement of the neovascular membrane area using ImageJ software. The image showcases the application of ImageJ software for precise delineation and measurement of the area occupied by the neovascular membrane.

The membrane removal rate refers to the speed at which proliferative membranes are excised during minimally invasive vitrectomy in patients with PDR. It is typically quantified as the ratio of the membrane area removed to the time required for removal. This ratio is known as the membrane removal rate and is expressed as the number of times the disk area is covered by proliferative membranes per minute. Initially, we employed ImageJ, a powerful Java-based image-processing software developed by the National Institutes of Health (NIH), to delineate the optic disk and membrane areas. By measuring the membrane area as a multiple (X) of the optic disk area, the membrane removal efficiency was calculated as (X ^*^ the disk area covered by the membranes)divided by [the peeling time (min)] (Figure 2).

#### 2.4.4. Intraoperative complications

Intraoperative complications such as iatrogenic retinal tears, inadvertent retinal or vascular injury due to intraocular instrument manipulation, and lens damage caused by instruments were documented by reviewing the surgical videos.

### 2.5. Assessment of surgical team satisfaction

We conducted a subjective satisfaction survey of participants who used the 3D surgical video system, including surgeons, surgical assistants, circulating nurses, and observers. All members of the surgical team completed a satisfaction questionnaire on the 3D surgical video system. The questionnaire focused on comparing and rating aspects such as resolution, stereoscopic effect, field of view, ergonomics, and comfort between the 3D video system and the traditional microscope. The ratings were provided on a scale of 1–5, where 1 = strongly disagree, 2 = disagree, 3 = no significant difference, 4 = agree, and 5 = strongly agree.

### 2.6. Statistical analysis

Statistical analysis was conducted using IBM SPSS Statistics for Windows, version 23.0 (IBM Corp., Armonk NY, USA). Continuous variables such as BCVA, IOP, surgical time, proliferative membrane removal time, proliferative membrane removal rate, normality, and homogeneity of variance were assessed. Differences between the two groups were compared by conducting t-tests or non-parametric tests (Mann–Whitney U test). Categorical variables such as gender, presence of intraoperative complications, and postoperative follow-up rate were compared by conducting chi-squared or Fisher's exact tests. Age is presented as the mean ± standard deviation, while other continuous variables are presented as the means ± standard error. A significance level of *p* < 0.05 was considered statistically significant.

## 3. Results

### 3.1. Clinical characteristics

The present study included 30 patients. While the distribution of operated eyes differed between the two groups, these differences were not clinically significant. Preoperatively, there were no statistically significant differences in age, BCVA, or IOP between the two groups.

Regarding the PDR classification, the 3D video surgery group included 10 cases of combined vitreous hemorrhage and four cases of combined tractional retinal detachment. The traditional microscope group included 11 cases of combined vitreous hemorrhage and five cases of combined tractional retinal detachment (*P* > 0.05).

In terms of follow-up, both groups had follow-up rates of 100% at 1 day and 1 week (p > 0.05). At 1 and 3 months, the follow-up rates were 92.8% for the 3D video surgery group and 87.5% for the traditional microscope group (p = 0.552) (Table 1).

**Table 1:** Preoperative statistics of PDR minimally invasive vitreous surgery using 3D surgical video system and traditional microscope.

	**3D surgical video group**	**Traditional microscope control group**	***P*-value**
Number of patients	14	16	
Age (Mean ± SD)	53.8 ± 15.6	53.3 ± 14.6	0.073
**Gender**			
Male	10	10	0.450
Female	4	6	
**Operated eye**			
Left	3	11	0.426
Right	11	5	
Vitreous/retinal hemorrhage	10	11	0.596
Tractional retinal detachment	4	5	
Preoperative BCVA	0.67 ± 0.55	0.33 ± 0.27	0.11
Preoperative IOP	15.53 ± 12.09	15.22 ± 7.36	0.736
**Follow-up rate**			
1D	100%	100%	1.000
1W	100%	100%	1.000
1M	92.8%	87.5%	0.552
3M	92.8%	87.5%	0.552

This table compares patient data for PDR minimally invasive vitreous surgery using 3D surgical video system and traditional microscope. The preoperative conditions and postoperative follow-up of 14 patients in the 3D surgical video group and 16 patients in the traditional microscope control group were compared. SD, Standard deviation; BCVA, Best corrected visual acuity; IOP, Intraocular pressure; 1D:1 day; 1W, 1 week; 1M, 1 month; 3M, 3 months.

### 3.2. Quantitative analysis of the intraoperative data

Our comparative analysis of vitreoretinal surgery using a 3D surgical video system and the traditional microscope in patients with PDR focused on four key quantitative indicators: total surgical time, membrane removal time, membrane removal rate, and intraoperative complications.

The average total surgical times for the 3D surgical video system and the traditional microscope groups were 30.25 ± 14.43 and 38.56 ± 18.71 mins, respectively (*p* = 0.051). Although the difference in the total surgical time between the two groups was not statistically significant, we observed a slight trend toward a shorter surgical time in the 3D surgical video system group, suggesting that the system may have certain advantages in terms of operational efficiency.

The average time for membrane removal was significantly shorter in the 3D surgical video system group than in the traditional microscope group (2.53 ± 1.52 vs. 3.23 ± 1.76 mins). Therefore, the use of the 3D surgical video system allowed faster membrane removal, thereby saving surgical time.

The average membrane removal rate was 0.55 ± 0.07 in the 3D surgical video system group and 0.41 ± 0.11 in the traditional microscope group. Further statistical analysis confirmed that the membrane removal rate in the 3D surgical video system group was significantly higher than that in the traditional microscope group. Therefore, the use of a 3D surgical video system enabled more effective removal of proliferative membranes, thereby enhancing surgical precision and efficiency.

Regarding intraoperative complications, we compared the occurrence of iatrogenic retinal breaks, retinal hemorrhage, and lens injury between the 3D surgical video system and traditional microscope groups. The results showed no iatrogenic retinal breaks in the 3D surgical video system group, while there was one case recorded in the traditional microscope group. There were three and five cases of retinal hemorrhage in the 3D surgical video system and traditional microscope groups, respectively. None of the groups reported lens injury. The complications did not differ significantly between the two groups. Thus, the use of the 3D surgical video system did not increase the risk of intraoperative complications and demonstrated excellent safety when compared with the traditional microscope (Table 2).

**Table 2:** Comparison of intraoperative data between 3D surgery video system group and traditional microscope system group for PDR minimally invasive vitrectomy surgery.

	**3D surgical video group**	**Traditional microscope system group**	***P*-value**
Surgery time (min)	30.25 ± 14.43	38.56 ± 18.71	0.051
Membrane peeling time (min)	2.53 ± 1.52	3.23 ± 1.76	0.042
Membrane peeling rate	0.55 ± 0.07	0.41 ± 0.11	0.018
(Area of optic disc/min)			
Intraoperative complications			
Iatrogenic retinal break	0	1	0.533
Vitreous hemorrhage	3	5	0.426
Lens injury	0	0	1.000

### 3.3. Postoperative follow-up and surgical team satisfaction

Evaluation of the improvements in postoperative visual acuity revealed significant improvements in visual acuity after surgery in both the 3D surgical video system and traditional microscope groups. Specifically, at 1 week, 1 month, and 3 months postoperatively, the BCVA values in the 3D surgical video system group were 0.45 ± 0.32, 0.29 ± 0.36, and 0.27 ± 0.31, respectively, and 0.47 ± 0.29, 0.33 ± 0.27, and 0.29 ± 0.36, respectively, in the traditional microscope group. Although the differences between the two groups were not statistically significant, these data demonstrated the success of the surgery and a favorable trend in postoperative recovery.

We observed changes in IOP during the postoperative follow-up period. Notably, we observed no statistically significant differences in the changes in IOP between the groups. This indicated that the use of the 3D surgical video system did not affect postoperative IOP control and exhibited stable and reliable performance in postoperative IOP management when compared with the traditional microscope.

Finally, we evaluated postoperative complications, including high IOP, vitreous hemorrhage, and retinal detachment. No statistically significant differences were observed between the two groups concerning these complications (*p* > 0.05). Moreover, no cases of retinal detachment were observed within the 3-month postoperative follow-up period in the 3D surgical video system group (Table 3).

**Table 3:** Comparison of postoperative outcomes between the 3D surgical video system group and the traditional microscope system group in PDR minimally invasive vitrectomy.

	**3D surgical video group**	**Traditional microscope system group**	***P*-value**
**Postoperative BCVA**			
1W	0.45 ± 0.32	0.47 ± 0.29	0.08
1M	0.29 ± 0.36	0.33 ± 0.27	0.12
3M	0.27 ± 0.31	0.29 ± 0.36	0.23
**Postoperative IOP**			
1W	19.23 ± 12.32	19.62 ± 13.09	0.18
1M	17.62 ± 11.53	16.53 ± 10.09	0.23
3M	15.02 ± 12.09	15.93 ± 11.03	0.27
Postoperative high intraocular pressure	3	4	0.581
Vitreous hemorrhage	1	2	0.552
Retinal detachment	0	1	0.533

BCVA, best corrected visual acuity; IOP, intraocular pressure; 1D, 1 day; 1W, 1 week; 1M, 1 month; 3M, 3 months.

We conducted a subjective satisfaction survey of participants who used the 3D surgical video system, including surgeons, surgical assistants, circulating nurses, and observers. We compared the performances of the 3D surgical video system and traditional microscope in several respects. The results showed that participants conducting procedures using the 3D surgical video system generally believed that the system exhibited significant advantages in image resolution, depth perception, ergonomics, and instructional presentation. They reported that the system provided clearer and more detailed images, which aided in more accurate observation and manipulation. The system also offered better depth perception, enabling more precise and intuitive surgical procedures. Additionally, the use of the 3D surgical video system reduced hand fatigue, providing a more comfortable working environment for the surgical operator, and better demonstrated the surgical steps and technical details, thereby facilitating training and education. However, no significant differences were observed between the two groups in terms of the remaining parameters, indicating that the performances of the 3D surgical video system and traditional microscope groups were relatively similar in these aspects (Table 4).

**Table 4:** Comparison of satisfaction survey between 3D surgical video system group and traditional microscope system group.

	**3D surgical video group**	**Traditional microscope system group**	***P*-value**
Image resolution	9.56 ± 0.73	7.44 ± 1.51	0.034
**Stereo perception**			
Low magnification	8.91 ± 0.95	8.41 ± 1.12	0.143
High magnification	9.00 ± 0.71	7.78 ± 0.97	0.041
Field of view	9.06 ± 2.43	9.34 ± 0.71	0.236
Image delay	8.10 ± 0.78	9.75 ± 1.84	0.083
Ergonomic	9.25 ± 0.72	5.00 ± 2.15	0.012
Shoulder and neck pain	6.44 ± 0.73	6.00 ± 1.12	0.237
Headache eye fatigue	5.52 ± 1.29	8.50 ± 2.20	0.019
Operational proficiency	9.06 ± 2.43	9.34 ± 0.71	0.763
Teaching demonstration	9.51 ± 0.72	5.81 ± 1.78	0.008
Total	9.37 ± 0.88	8.12 ± 1.59	0.042

## 4. Discussion

This study investigated the safety and effectiveness of integrating 3D surgical video systems in PDR surgery, as compared to traditional microscope–assisted surgery. The comparison focused on various aspects, such as surgical duration; intraoperative iatrogenic injuries, such as iatrogenic retinal tear, retinal hemorrhage, and lens damage; postoperative outcomes, such as BCVA and IOP; and postoperative complications, such as elevated IOP, vitreous hemorrhage, and retinal detachment.

The results confirmed the safety and effectiveness of PDR surgery using a 3D surgical video system. In the traditional microscope–assisted group, one patient experienced an iatrogenic retinal tear, and another patient developed recurrent retinal detachment postoperatively. The tight adhesion between the proliferative membrane and the retina, which makes it difficult to distinguish between the membrane and normal retinal structures, may have contributed to the challenges in tissue layer separation. Additionally, a prolonged disease course and severity could also be factors. However, no severe complications were observed in the 3D surgical video group.

In the landscape of vitreoretinal surgery, the application of 3D surgical video systems has garnered attention both domestically and internationally, with studies illustrating their effectiveness in addressing retinal and vitreous disorders (15). Notably, pioneers such as Eckardt, Kita, and others laid the foundation by introducing 3D surgical video systems to vitreoretinal surgery. Additionally, comparisons between the 3D surgical video and traditional microscope-based systems have been conducted, indicating similar surgical durations and postoperative outcomes (14, 16–19). Our study contributes to this evolving field by introducing a novel concept, the “stripping efficiency.” This concept demonstrates the distinct advantages of utilizing a 3D surgical video system in proliferative diabetic retinopathy surgeries. By focusing on the efficiency aspect, we delve into a unique dimension that sheds light on the tangible benefits of this technology. Moreover, our study employs a questionnaire-based approach to gather insights from both surgeons and observers who experienced surgeries using the 3D surgical video system. This method not only provides valuable quantitative data but also offers qualitative perspectives on the usability and effectiveness of the system. This assessment underscores the comprehensive nature of our evaluation.

Objective data indicators were employed to further confirm the advantages of the 3D surgical video system in PDR in terms of depth perception and image resolution. The efficiency of proliferative membrane removal was compared with the rate of membrane removal, represented as x times the disk area removed per minute. The 3D surgical video system group exhibited statistically significant differences in membrane removal time and removal rate when compared with the traditional microscope–assisted group. Additionally, a subjective satisfaction evaluation questionnaire was developed and administered to 18 surgical team members. At low magnification, most agreed that the depth perception and resolution were similar between the 3D surgical video system and the traditional microscope system. However, at high magnifications during intraocular procedures, the 3D system was considered to offer significantly superior depth perception and resolution. These excellent parameters provided by the 3D surgical video system enhanced 3D visualization of the intraocular anatomy, leading to more precise intraoperative manipulation. This effectively reduced the frequency of intraocular instrument insertion and withdrawal, thereby mitigating the risk of retinal hemorrhage and iatrogenic retinal tears caused by mechanical manipulation during surgery. Freeman et al. verified the visual performance of a 3D surgical video system using subjective and objective data (20–23).

However, this study had a limited sample size for membrane peeling. Obtaining a larger sample size and integrating artificial intelligence (AI) technology for automatic delineation and measurement of the proliferative membrane area would allow more accurate and meaningful calculations of membrane removal efficiency. The precision of area calculations in the peripheral retina is a challenge due to various factors, including image distortion, variable magnification, and potential inaccuracies in capturing fine details. While our methodology was designed to address these challenges to the best extent possible, it is important to recognize that there may be inherent limitations in accurately quantifying peripheral retinal areas. Future studies could explore advanced imaging techniques to enhance the precision of peripheral retinal area measurements.

Furthermore, in the questionnaire survey, the participants expressed a positive evaluation bias toward image resolution, depth perception, field of view, and educational demonstration of the 3D surgical video system. They stated that wearing 3D polarized glasses provided simultaneous, synchronous, and stereoscopic surgical images, allowing a better understanding of the intraocular anatomy and surgical steps for both surgical participants and beginners. However, with increasing surgery duration and number of surgeries, little subjective difference was observed between the 3D surgical video system and the traditional microscope in terms of proficiency, ergonomics, and image latency. Nevertheless, the paradigm-shifting seated and upright viewing posture of the 3D video system eliminated the need for surgeons to bend their heads for prolonged periods, thereby reducing the burden on the surgeons' necks and shoulders and alleviating fatigue and discomfort during surgery.

In conclusion, this study's results confirmed that the 3D surgical video system enabled clearer visualization of pathological tissues and differentiation between proliferative membranes and normal retinal tissues during minimally invasive PDR vitrectomy. This allowed surgeons to perform membrane peeling with greater precision and speed, effectively relieving the traction caused by the proliferative membrane and vitreous in the retina. This effect reduced mechanical traction injury caused by repeated membrane peeling and improved the visual prognosis in some patients. The 3D surgical video system also demonstrated safety and effectiveness in PDR surgery, leading to more precise, stable, efficient, and safe PDR surgical treatment. However, this study had a limited sample size and a short follow-up duration; therefore, further research with a larger sample size is needed to investigate the application effects of the 3D surgical video system in minimally invasive PDR surgery.

## Data availability statement

The original contributions presented in the study are included in the article/supplementary material, further inquiries can be directed to the corresponding author.

## Ethics statement

The studies involving humans were approved by the Ethics Committee of Tianjin Medical University Eye Hospital (Approval No. 2022JS-01). The studies were conducted in accordance with the local legislation and institutional requirements. Written informed consent for participation was not required from the participants or the participants' legal guardians/next of kin in accordance with the national legislation and institutional requirements.

## Author contributions

BH, XZ, DZ, and WL conceived and designed the study, conducted the experiments, analyzed the data, and wrote the manuscript. HH and ZN assisted in the study design, provided guidance throughout the research process, and contributed to the writing and editing of the manuscript. HG contributed to the data analysis and interpretation of the results, and critically reviewed the manuscript. ZW conducted the literature review, performed statistical analyses, and assisted in manuscript preparation. XL provided technical support and assistance in the data collection and analysis. All authors have read and approved the final version of the manuscript and agree to be accountable for all aspects of the study.

## References

[1] Yau JW Rogers SL Kawasaki R Lamoureux EL Kowalski JW Bek T. Global prevalence and major risk factors of diabetic retinopathy. Diabetes Care. (2012) 35:556–64. 10.2337/dc11-1909 22301125 PMC3322721

[2] Lin KY Hsih WH Lin YB Wen CY Chang TJ. Update in the epidemiology, risk factors, screening, and treatment of diabetic retinopathy. J Diabetes Investig. (2021) 12:1322–25. 10.1111/jdi.13480 33316144 PMC8354492

[3] Sabanayagam C Banu R Chee ML Lee R Wang YX Tan G. Incidence and progression of diabetic retinopathy: a systematic review. Lancet Diabetes Endocrinol. (2019) 7:140–9. 10.1016/S2213-8587(18)30128-1 30005958

[4] Chaudhary S Zaveri J Becker N. Proliferative diabetic retinopathy (PDR). Dis Mon. (2021) 67:101140. 10.1016/j.disamonth.2021.101140 33546872

[5] Berrocal MH Acaba LA Acaba A. Surgery for diabetic eye complications. Curr Diab Rep. (2016) 16:99. 10.1007/s11892-016-0787-6 27612846

[6] Brodie FL Seider MI. Subacute vision loss in a young pregnant patient with proliferative diabetic retinopathy. JAMA Ophthalmol. (2018) 136:948–9. 10.1001/jamaophthalmol.2017.6760 29799966

[7] Gross JG Glassman AR. A novel treatment for proliferative diabetic retinopathy: anti-vascular endothelial growth factor therapy. JAMA Ophthalmol. (2016) 134:13–4. 10.1001/jamaophthalmol.2015.5079 26583372

[8] Gross JG Glassman AR Liu D Sun JK Antoszyk AN Baker CW. Five-year outcomes of panretinal photocoagulation vs intravitreous ranibizumab for proliferative diabetic retinopathy: a randomized clinical trial. JAMA Ophthalmol. (2018) 136:1138–48. 10.1001/jamaophthalmol.2018.3255 30043039 PMC6233839

[9] Schreur V Brouwers J Van Huet RAC Smeets S Phan M Hoyng CB. Long-term outcomes of vitrectomy for proliferative diabetic retinopathy. Acta Ophthalmol. (2021) 99:83–9. 10.1111/aos.14482 32643273 PMC7891313

[10] Zheng CZ Ren XJ Ke YF Wen DJ Li XR. [Minimally invasive vitrectomy for the treatment of severe proliferative diabetic retinopathy]. Zhonghua Yan Ke Za Zhi. (2021) 57(6):440-446. 10.3760/cma.j.cn112142-20200812-00538 34098693

[11] Wang Y Zhao X Zhang W Yang J Chen Y. Three-dimensional head-up display versus standard operating microscope for vitrectomy surgery: a systematic review and meta-analysis. Retina. (2022) 42:1151–60. 10.1097/IAE.0000000000003414 35067608

[12] Zhang Z Wang L Wei Y Fang D Fan S Zhang S. The preliminary experiences with three-dimensional heads-up display viewing system for vitreoretinal surgery under various status. Curr Eye Res. (2019) 44:102–9. 10.1080/02713683.2018.1526305 30265818

[13] Palácios RM de Carvalho ACM Maia M Caiado RR Camilo DAG Farah ME. An experimental and clinical study on the initial experiences of Brazilian vitreoretinal surgeons with heads-up surgery. Graefes Arch Clin Exp Ophthalmol. (2019) 257:473–83. 10.1007/s00417-019-04246-w 30645695

[14] Eckardt C Paulo EB. Heads-up surgery for vitreoretinal procedures: an experimental and clinical study. Retina. (2016) 36:137–47. 10.1097/IAE.0000000000000689 26200516

[15] Zhang T Tang W Xu G. Comparative analysis of three-dimensional heads-up vitrectomy and traditional microscopic vitrectomy for vitreoretinal diseases. Curr Eye Res. (2019) 44:1080–6. 10.1080/02713683.2019.1612443 31021174

[16] Zhao XY Zhao Q Li NN Meng LH Zhang WF Wang EQ. Surgery-related characteristics, efficacy, safety and surgical team satisfaction of three-dimensional heads-up system versus traditional microscopic equipment for various vitreoretinal diseases. Graefes Arch Clin Exp Ophthalmol. (2023) 261:669–79. 10.1007/s00417-022-05850-z 36210375 PMC9988774

[17] Asani B Siedlecki J Schworm B Mayer WJ Kreutzer TC Luft N Priglinger SG. 3D heads-up display vs. standard operating microscope vitrectomy for rhegmatogenous retinal detachment. Front Med. (2020) 7:615515. 10.3389/fmed.2020.615515 33415120 PMC7782350

[18] Weinstock RJ Diakonis VF Schwartz AJ Weinstock AJ. Heads-up cataract surgery: complication rates, surgical duration, and comparison with traditional microscopes. J Refract Surg. (2019) 35:318–22. 10.3928/1081597X-20190410-02 31059581

[19] Liu J Wu D Ren X Li X. Clinical experience of using the NGENUITY three-dimensional surgery system in ophthalmic surgical procedures. Acta Ophthalmol. (2021) 99:e101–8. 10.1111/aos.14518 32643263

[20] Ehlers JP Uchida A Srivastava SK. The integrative surgical theater: combining intraoperative optical coherence tomography and 3d digital visualization for vitreoretinal surgery in the discover study. Retina. (2018) 38:S88–s96. 10.1097/IAE.0000000000001999 29256988 PMC6005714

[21] Freeman WR Chen KC Ho J Chao DL Ferreyra HA Tripathi AB. Resolution, depth of field, and physician satisfaction during digitally assisted vitreoretinal surgery. Retina. (2019) 39:1768–1771. 10.1097/IAE.0000000000002236 29965938 PMC6310113

[22] Babu N Kohli P Jena S Ramasamy K. Utility of digitally assisted vitreoretinal surgery systems (DAVS) for high-volume vitreoretinal surgery centre: a pilot study. Br J Ophthalmol. (2020) 104:432–6. 10.1136/bjophthalmol-2019-314123 31177188

[23] González-Saldivar G Chow DR. Optimizing visual performance with digitally assisted vitreoretinal surgery. Ophthalmic Surg Lasers Imaging Retina. (2020) 51:S15–s21. 10.3928/23258160-20200401-02 32348530

